# Agreement between the GAITRite^®^ System and the Wearable Sensor BTS G-Walk^®^ for measurement of gait parameters in healthy adults and Parkinson’s disease patients

**DOI:** 10.7717/peerj.8835

**Published:** 2020-05-22

**Authors:** Slávka Vítečková, Hana Horáková, Kamila Poláková, Radim Krupička, Evžen Růžička, Hana Brožová

**Affiliations:** 1 Faculty of Biomedical Engineering, Czech Technical University in Prague, Kladno, Czech Republic; 2 Department of Neurology and Center of Clinical Neuroscience, 1st Faculty of Medicine and General University Hospital in Prague, Charles University, Prague, Czech Republic

**Keywords:** Gait analysis, Wearable sensors, Biomechanics, Parkinson’s disease

## Abstract

**Background:**

Nowadays, the most widely used types of wearable sensors in gait analysis are inertial sensors. The aim of the study was to assess the agreement between two different systems for measuring gait parameters (inertial sensor vs. electronic walkway) on healthy control subjects (HC) and patients with Parkinson’s disease (PD).

**Methods:**

Forty healthy volunteers (26 men, 14 women, mean age 58.7 ± 7.7 years) participated in the study and 24 PD patients (19 men, five women, mean age 62.7 ± 9.8 years). Each participant walked across an electronic walkway, GAITRite, with embedded pressure sensors at their preferred walking speed. Concurrently a G-Walk sensor was attached with a semi-elastic belt to the L5 spinal segment of the subject. Walking speed, cadence, stride duration, stride length, stance, swing, single support and double support phase values were compared between both systems.

**Results:**

The Passing-Bablock regression slope line manifested the values closest to 1.00 for cadence and stride duration (0.99 ≤ 1.00) in both groups. The slope of other parameters varied between 0.26 (double support duration in PD) and 1.74 (duration of single support for HC). The mean square error confirmed the best fit of the regression line for speed, stride duration and stride length. The y-intercepts showed higher systematic error in PD than HC for speed, stance, swing, and single support phases.

**Conclusions:**

The final results of this study indicate that the G-Walk system can be used for evaluating the gait characteristics of the healthy subjects as well as the PD patients. However, the duration of the gait cycle phases should be used with caution due to the presence of a systematic error.

## Introduction

Measurements of temporo-spatial gait parameters are used by physicians and physiotherapists for diagnosing gait disorders, disease progression monitoring and controlling the effect of different therapeutic methods (Nonnekes et al., 2018). Various types of wearable or non-wearable systems have been used by researchers or rehabilitation professionals for gait analysis. However, non-wearable sensors are limited to the controlled research facilities. Wearable sensors, on the other hand, allow to analyse the gait even outside the laboratory in everyday activities (Muro-de-la Herran, Garcia-Zapirain & Mendez-Zorrilla, 2014).

For the last decade, the GAITRite system was considered as “the gold standard” in gait parameters measurement and gait analysis (Van Uden & Besser, 2004; Menz et al., 2004; Webster, Wittwer & Feller, 2005; Heikkilä et al., 2017; Jagos et al., 2017). This system has been mentioned in more than 400 studies in PubMed. It is often used as the reference system for comparison with other types of measurement systems (Jagos et al., 2017). The validity and test-retest reliability of this system has been studied on healthy subjects (Bilney, Morris & Webster, 2003; Van Uden & Besser, 2004) as well as in people suffering from neurodegenerative or musculoskeletal disorders, e.g., Parkinson‘s disease (Rennie et al., 2017) or patients with a total knee replacement (Heikkilä et al., 2017), etc. On the other hand, there are some limitations to the GAITRite system: the examination mat needs a lot of space and is heavy for manipulation. Furthermore, the initial cost is relatively high.

Nowadays, the most widely used types of sensors in gait analysis are inertial sensors. The inertial sensors use a combination of an accelerometer, gyroscope and sometimes a magnetometer (Muro-de-la Herran, Garcia-Zapirain & Mendez-Zorrilla, 2014). An accelerometer works on the fundamentals of Newton’s Laws of Motion, it is a type of wearable inertial sensor that can measure acceleration along its sensitive axis. The gyroscope, an angular velocity sensor which also uses the principles of a magnetometer, is based on magnetoresistive effects (Tao et al., 2012). The inertial sensor for the G-Walk has been used in studies for measuring the gait parameters of older adult (Pau et al., 2014), patients with hereditary cerebellar ataxias (Leonardi et al., 2017) or Parkinson’s disease (Zago et al., 2018). The G-Walk for gait analysis in healthy subjects was documented as reliable for all measured spatiotemporal parameters (i.e., speed, cadence, stride length, stride duration, single support, double support, swing duration, stance duration) and valid for speed, cadence, stride length, and stride duration (De Ridder et al., 2019; Park & Woo, 2015). Wearable systems for gait measurement are lightweight, portable, relatively cheap and easy to use. Nevertheless, the use of inertial sensors has certain disadvantages. Analysis of the signals obtained from inertial sensors is computationally complex for estimating gait parameters and is susceptible to noise and interference of external factors, which are uncontrolled by specialist. The wireless connection recording device has a great demand on power consumption, limiting the time it can be used by the battery duration. Also placing the devices on the subject’s body may be uncomfortable (Muro-de-la Herran, Garcia-Zapirain & Mendez-Zorrilla, 2014) and can contribute to difficulties, especially in the case of a tremor.

The aim of this study was to assess the agreement between the two systems for measurement of temporal and spatial gait parameters in healthy adults and Parkinson’s disease (PD) patients. In our study, we used the GAITRite as the reference system to the new wearable inertial system G-Walk in two subject groups.

## Methods

### Participants

Forty healthy volunteers (26 men, 14 women, age range from 43 to 71 years; mean age 58.7 ± 7.7 years; weight 85.5 ± 18.4 kg; height 176.0 ± 8.4 cm) and 24 PD patients (19 men, 5 women, age range from 42 to 78 years; mean age 62.7 ± 9.8 years; weight 83.8 ± 17.4 kg, height 174.7 ± 6.7 cm, disease duration 8.3 ± 4.5 years, L-dopa equivalent 1033,6 ± 588,6 mg (Tomlinson et al., 2010) participated in the study. The inclusion criterion for this study from healthy volunteers was between the age of 18 and 75 years. For the patient group, the inclusion criterion was a diagnosis of PD. The exclusion criteria were any subjective complaint of an abnormal gait, any other neurological or orthopaedic condition that might affect gait, and an alcohol or drug addiction. All PD patients were examined using the Movement Disorder Society Unified Parkinson’s Disease Rating Scale (MDS-UPDRS), from which the subscores were calculated: UPDRS I 8.0 ± 5.0, UPDRS II 11.8 ± 7.0, UPDRS III 22.4 ± 13.5 (in the ON state before testing), UPDRS IV 4.8 ± 4.2. The Hoehn&Yahr score of the PD participants was 2.1 ± 0.5.

This study was approved by the Ethical Committee of General University Hospital in Prague (IRB approval number: 132/15) and a written informed consent was obtained from all participants before entering the study.

### Equipment

The GAITRite^^®^^ system is a special roll-up carpet for measuring temporal and spatial gait parameters. This electronic walkway is connected to a USB port for a computer with GAITRite software (CIR Systems Inc. Clifton, NJ, USA). A standard GAITRite electronic walkway contains eight sensor pads encapsulated in a roll-up carpet that represents an active area of 61 cm wide and 488 cm long. In this arrangement there are a total of 18,432 sensors on the active area of a grid (grid pattern 48 × 384, sensors are placed 1.27 cm apart). As the patient walks across the carpet, the system continuously scans the sensors to detect objects. The system is portable, and no devices need to be attached to the patient.

The BTS G-Walk^^®^^ (G-Sensor 2) is a portable, wireless, inertial system with wearable sensors. It weighs 37 g and its dimensions are 70 × 40 × 18 mm. This device is composed of a triaxial accelerometer (16 bit/axes) with multiple levels of sensitivity (±2, ±4, ±8, ±16 g), a triaxial gyroscope (16 bit/axes) with multiple levels of sensitivity (±250, ±500, ± 1000, ± 2000°/s) and a triaxial magnetometer (13 bit, ± 1,200 µT). The device is attached with a semi-elastic belt to the waistline of examined subject, it records the acceleration. All acceleration data was sampled at a 100 Hz frequency, transmitted by Bluetooth to a notebook and processed using the special software program BTS G-Studio (BTS Bioengineering S.p.A., Italy).

Compared systems employ different strategies to acquire data and calculate gait parameters. The basis for developing walking algorithms of the accelerometer has been described in previous studies (Zijlstra & Hof, 2003; Godfrey et al., 2015; Cimolin et al., 2017; Panebianco et al., 2018), however the exact algorithms for G-Walk are unknown, as this is an internal know-how of BTS Bioengineering. The extensive review and comparison of various systems utilized in gait analysis can be found elsewhere (Muro-de-la Herran, Garcia-Zapirain & Mendez-Zorrilla, 2014; Petraglia et al., 2019).

### Procedure

Testing was performed at the Department of Neurology, the First Faculty of Medicine, Charles University in Prague. The test consisted of walking at a preferred speed for a distance of 10 m. Each participant completed 2 successive passes across the GAITRite carpet with a G-Walk sensor, attached to the L5 spinal segment of the lower back using a semi-elastic belt. Used setting was following: accelerometer ±2g, magnetometer OFF, gyroscope ±2000°/s. Gait characteristics were measured by both systems simultaneously.

Each trial started 2 m in front of the carpet and finished 2 m beyond the carpet, so acceleration and deceleration occurred out of the borders of the electronic walkway. The participants were instructed to begin walking from a starting line on the command “Get ready –Attention –Go.”, and to continue walking until they reached the finish line placed 2 m beyond the end of the walkway. The lower limb length was measured for each participant and filled in for both G-Walk and GAITRite software (for step length measurement).

### Variable

Data from the two trials were averaged for each participant, to define the individual mean of speed, cadence or stride length. The parameters represented the average results of both sides. The average stance phase, swing phase, single support and double support phase duration were expressed as percentages of the gait cycle. The double support for the G-Walk was calculated as a sum of the first (initial) double support and the terminal double support.

Only entire steps obtained by GAITRite were taken for analyses and the corresponding steps captured by the G-Walk sensor.

### Statistical analysis

To assess the concurrent validity and potential bias of the G-Walk system we processed data in three steps. First, to determine a statistically significant difference between the mean values of both measurement systems, a paired sample *t*-test was used. The level of significance *α* was set at *0.05*. However, statistically non-significant differences did not imply concurrent validity. Besides statistically significant differences, the source of error should be analysed in more detail. For these reasons, we employed two more methods for comparing the two measurement instruments.

Second, we studied scatter plots, slope, intercept, and residuals of the regression line. We were interested in whether the gradient of the slope was equal to one (“y = x”). Theoretically, if the slope is equal to one and the *y-* intercept is equal to zero, then “y = x” (where GAITRite value is “x”, G-Walk value is “y”) (Zaki et al., 2012). The intercept and slope represent systematic errors in measurement. The intercept indicates a fixed amount of error present in the measurement, whereas the slope is a sample of proportional error. The residuals represent random differences. We used the Passing-Bablok regression analysis (Passing & Bablok, 1983), because this method isn’t sensitive to distribution of errors and is robust against data outliers, therefore it is more suitable for data analyses than the linear regression model (the least-squares) (Bilić-Zulle, 2011). The Passing-Bablok method is valid only when a linear relationship exists between *x* and *y* (which can be confirmed by the cusum linearity test) (Passing & Bablok, 1983). To determine the extent to which the Passing-Bablok analysis fits the data, the mean square error (MSE) was used.

Finally, the intraclass correlation coefficient (ICC) has recently been used to evaluate the level of agreement between the G-Walk and the GAITRite (De Ridder et al., 2019). Although intraclass correlation coefficients have been reported as the most frequently used tool for validity assessment (Wolak, Fairbairn & Paulsen, 2012), they cannot answer whether measurement methods can be used interchangeably (Bland & Altman, 1990). To better compare with this previous study, we determined the ICC as well (type ICC (2, k)). The ICC values were interpreted as follows: >0.75 excellent, 0.40 to 0.75 fair to good, <0.40 poor agreement (Fleiss, Levin & Paik, 2013).

Matlab software (version 2015a) was used for statistical analysis.

## Results

The mean and standard deviation values for temporal and spatial gait parameters for both methods are shown in Table 1. We calculated the paired *t*-test *p*-value, repeatability coefficients, and slopes for regression equations (Passing-Bablok) with 95% confidence intervals along with y-intercepts (see Table 2). Paired *t*-test showed a statistically significant difference in a half of all analyzed parameters for both subject groups. Only cadence, speed, stride length and stride duration did not exhibit a *p*-value less than 0.05. The Passing-Bablock regression line slope manifested the values closest to 1.00 for cadence and stride duration (0.99 ≤ X ≤ 1.00) in both groups. The slope of other parameters varied between 0.26 (double support duration in PD) and 1.74 (duration of single support for HC). The MSE revealed a significantly lower error for cadence in HC than in PD. The temporal parameters (stance duration, swing duration, double support, single support) exhibit significantly higher error in HC than in PD. The MSE of other parameters in PD are similar to those in HC. The ICC demonstrated excellent reliability for speed, cadence, stride duration, and stride length (rho > 0.75) in both groups.

**Table 1:** Descriptive statistics of analyzed data. Mean and standard deviation (in brackets) are provided.

	**HA**		**PD**	
	**GAITRite**	**G-Walk**	**GAITRite**	**G-Walk**
**Cadence (steps/min)**	110.84 (10.35)	111.01 (10.52)	106.86 (14.28)	106.73 (14.35)
**Speed (m/s)**	1.38 (0.21)	1.34 (0.24)	1.10 (0.36)	1.06 (0.30)
**Stride duration (s)**	1.09 (0.10)	1.10 (0.10)	1.18 (0.26)	1.17 (0.19)
**Stride length (m)**	1.49 (0.15)	1.45 (0.21)	1.22 (0.30)	1.18 (0.24)
**Stance (%)**	63.47 (1.83)	60.24 (2.09)	65.70 (3.86)	59.11 (2.02)
**Swing (%)**	36.54 (1.83)	39.76 (2.09)	34.31 (3.86)	40.89 (2.02)
**Double support (%)**	26.76 (3.85)	20.45 (4.25)	31.90 (7.93)	18.42 (4.13)
**Single support (%)**	36.41 (1.82)	39.83 (2.20)	34.38 (3.85)	40.74 (2.23)

**Notes.**

HA Healthy adults PD Patients with Parkinson’s disease

**Table 2:** The assessment of concurrent validity between two gait measurement instruments and an analysis of error. The results of inter-instruments agreement and error of measurement in group of healthy adults (HA) and patients with Parkinson’s disease (PD). Paired *t*-test *p*-value, intraclass correlation coefficient (ICC) and Passing-Bablok (PB) slope, intercept and residuals mean are reported.

	**Paired t-test: *p*-value**		**ICC(2,k): rho (95% CI)**		**PB: slope (95% CI)**		**PB: intercept (95% CI)**		**PB: residuals mean (1.96 RSD)**		**PB: MSE**	
**Gait variable**	**HA**	**PD**	**HA**	**PD**	**HA**	**PD**	**HA**	**PD**	**HA**	**PD**	**HA**	**PD**
**Cadence (steps/min)**	0.636	0.871	0.99 (0.97,0.99)	0.98 (0.96,0.99)	0.97 (0.92,1.03)	0.97 (0.90,1.04)	2.98 (−2.69,9.59)	2.41 (−4.32,11.21)	0.08 (±4.34)	0.53 (±7.75)	4.78	15.28
**Speed (m/s)**	0.103	**0.049**	0.87 (0.76,0.93)	0.98 (0.94,0.99)	1.02 (0.81,1.31)	0.86 (0.77,0.94)	−0.06 (−0.43,0.21)	0.11 (0.03,0.20)	−0.01 (±0.29)	<0.01 (±0.13)	0.02	<0.01
**Stride duration (s)**	0.134	0.689	0.98 (0.97,0.99)	0.81 (0.56,0.92)	0.99 (0.92,1.05)	1.00 (0.94,1.06)	0.02 (−0.05,0.09)	0.01 (−0.06,0.07)	<0.01 (±0.05)	−0.02 (±0.36)	<0.01	0.03
**Stride length (m)**	0.113	0.191	0.82 (0.67,0.91)	0.95 (0.89,0.98)	1.50 (1.17,2.10)	0.80 (0.67,0.92)	−0.76 (−1.63,−0.28)	0.21 (0.05,0.36)	−0.02 (±0.30)	<0.01 (±0.16)	0.02	0.01
**Stance (%)**	**<0.001**	**<0.001**	0.22 (−0.30,0.58)	0.12 (−0.22,0.48)	1.49 (0.78,2.57)	0.36 (0.11,0.67)	−35.18 (−104.29,10.58)	35.89 (15.57,51.75)	0.68 (±5.74)	−0.45 (±4.20)	8.82	4.61
**Swing (%)**	**<0.001**	**<0.001**	0.22 (−0.30,0.58)	0.12 (−0.22,0.48)	1.52 (0.78,2.59)	0.36 (0.11,0.67)	−15.03 (−52.60,11.41)	28.17 (17.44,36.98)	−0.71 (±5.81)	−0.46 (±4.19)	9.09	4.60
**Double support (%)**	**<0.001**	**<0.001**	0.24 (−0.31,0.61)	0.10 (−0.22,0.45)	1.48 (0.86,2.60)	0.26 (0.03,0.60)	−20.97 (−52.90,−3.29)	10.76 (−0.24,17.83)	1.71 (±11.78)	−0.59 (±8.27)	38.12	17.40
**Single support (%)**	**<0.001**	**<0.001**	0.22 (−0.29,0.59)	0.13 (−0.24,0.50)	1.74 (0.94,3.28)	0.27 (0.01,0.71)	−22.96 (−78.50,5.60)	31.44 (16.16,40.27)	−0.63 (±6.38)	0.10 (±4.33)	10.72	4.69

**Figure 1:**
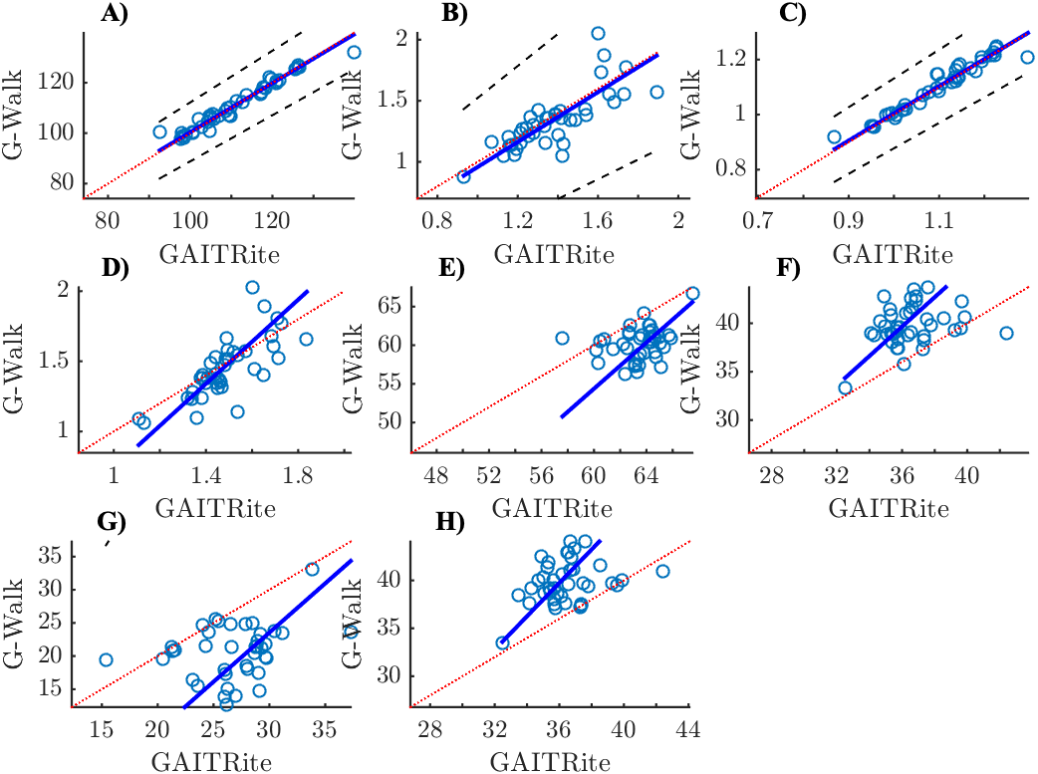
Scatter plots with Passing-Bablok regression lines and identity lines (*y* = *x*) for control group. (A) Cadence (step/min), (B) Speed (m/s), (C) Stride duration (s), (D) Stride lenght (m), (E) Stance (%), (F) Swing (%), (G) Double support (%), (H) Single support (%); *x*-axis: GAITRite, *y*-axis: G-Walk.

**Figure 2:**
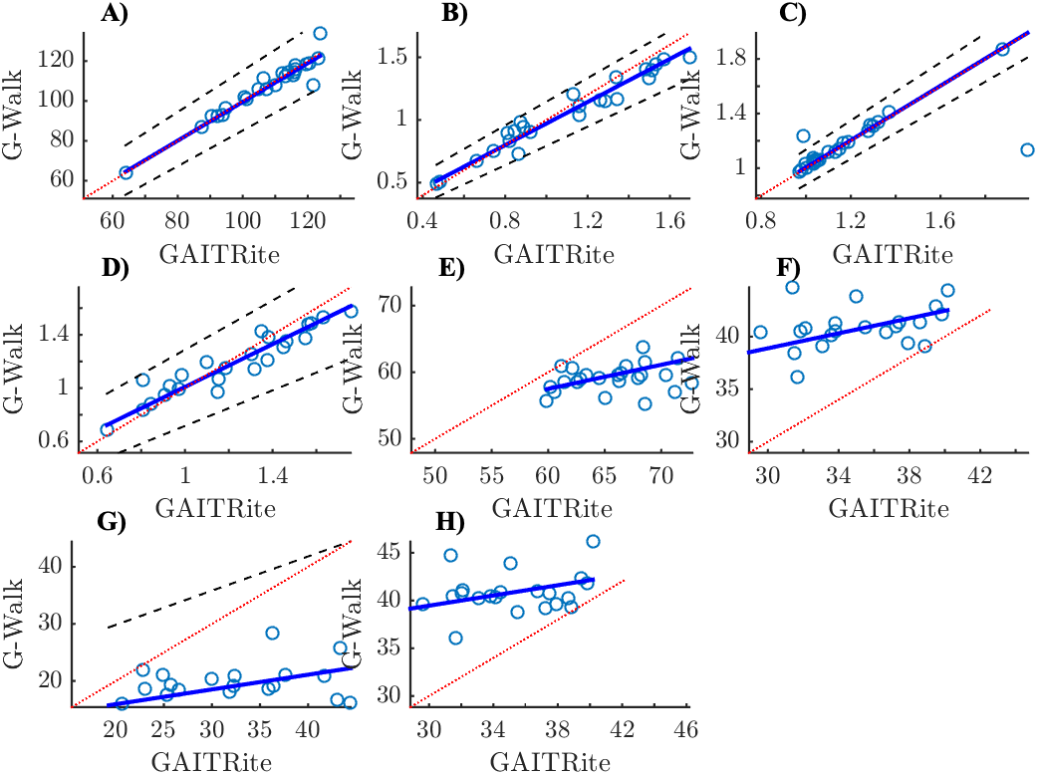
Scatter plots with Passing-Bablok regression lines and identity lines (*y* = *x*) for Parkinson’s disease patients. (A) Cadence (step/min), (B) Speed (m/s), (C) Stride duration (s), (D) Stride lenght (m), (E) Stance (%), (F) Swing (%), (G) Double support (%), (H) Single support (%); *x*-axis: GAITRite, *y*-axis: G-Walk.

Figures 1 and 2 show scatter plots with Passing-Bablok regression lines for all parameters (i.e., speed, cadence, stride length, stride duration, stance, swing, single support and double support phase).

## Discussion

We performed a study to assess the agreement between G-Walk and GAITRite as a reference system for measurement of temporal and spatial gait parameters in healthy adults and PD patients. The statistical analysis via *t*-test confirmed the hypothesis that the parameters measured by G-Walk and GAITRite are the same only for cadence, stride length and stride duration in both groups and in addition for speed in HC. The Passing-Bablok regression slope also showed agreement for cadence, speed, stride duration in both groups. For stride length the regression slope showed agreement only in PD. On the basis of the ICC (2, k) the results attempt to indicate that the G-Walk is a valid measurement tool for speed, cadence, stride duration and stride length. Although our results are similar to the ICC values achieved by other authors, who compared the two devices for measuring gait parameters (Bilney, Morris & Webster, 2003; DesJardins et al., 2016; De Ridder et al., 2019), these conclusions for speed and stride length are contradictory with the results of other employed methods. First, the paired *t*-test showed that values measured by both systems are statistically different. Second, Passing-Bablok regression slopes did not imply interchangeable values. The suitability of ICC for instrument validity assessment has recently been discussed and details can be found elsewhere (Vaz et al., 2013; Prescott, 2019).

It suggests that the values measured by G-Walk and GAITRite are interchangeable for these parameters: speed, cadence, stride duration and stride length.

For parameters which differed between the two systems (stance, swing, double support, single support) we performed an analysis of systematic and random error via intercepts and residuals, respectively. The results pointed that the systematic error influenced the discrepancy more between the systems than random error. The systematic error in the PD group was higher than the systematic error in HC group for almost all parameters.

As the stride duration provided by the G-Walk is comparable to GAITRite it can be deduced that the gait cycle is recognized properly. But the duration of the phases differentiates between systems. As the systematic error is present in phases duration, it can be concluded that detection of foot contact events should be improved. It should be noted that G-Walk uses trunk-based gait event detection. Trunk-based algorithms usually use peak or zero-crossing detection algorithms for heel strike and toe-off event identification (Panebianco et al., 2018). De Ridder et al. (2019) recently pointed out to inconsistency of temporal parameters based on toe-off event between G-Walk and GAITRite. Bravi et al. (2020) also noticed incorrect gait cycle phase recognition by G-Walk.

Recently, two other studies comparing G-Walk wearable system and pressure sensitive walkway were published. First, De Ridder et al. (2019) analyzed the spatiotemporal parameters of groups of healthy adults. The discrepancy between our results can be caused by the different ages of the participants. Therefore, ongoing studies should analyze the relationship between age and the amount of systematic error. Moreover, as the G-Walk is a proprietary system and its used algorithms are not published, the discrepancy may be caused by different versions of the G-Walk Studio. Hence, the version of the employed software should be reported in studies. Second, Park & Woo (2015) analyzed the correlation between spatiotemporal parameters computed by the G-Walk and the GAITRite systems. Owing to the different aim of this paper, our results are not comparable.

G-Walk unlike GAITRite calculates also tilt, obliquity and rotation of the pelvis, however we did not evaluate those parameters.

The comparison of two gait measurement systems presented here is demonstrated on two subject groups: healthy adults and PD patients. Although the procedure is general, the results are closely tied to these specific subject groups. Analysis of inertial sensor signals is computationally complex. The estimation of gait parameters can be affected by abnormal gait cycle (as the gait event detection relies on distinctive points). Moreover, it has been shown that the walking speed is potential factor affecting validity of wearable sensors (Greene et al., 2012). For example, patients with neurological disorder exhibit different gait patterns (e.g., PD patients show shuffling gait, in the hemiplegic gait is marked by abnormal initial contact during stance (McGee, 2012); slow gait is one of the most common indicators among post-acute acute myocardial infarction older adults (Dodson et al., 2012); depressed patients have gait manifested by an slowing of gait and weak lift-off of the heel (Sloman et al., 1982). As the different subject groups may be characterized by different gait pattern or speed, the validity analysis should be performed individually per group profile.

We must admit that this study contains certain limitations. First, the main limitation was that only healthy subjects and patients were only tested in their ON state. In future research we recommend including patients in the OFF state. Second, as the employed Passing-Bablok method quantifies systematic error between instruments in measurement units, the results cannot be directly compared across parameters. The magnitudes of intercepts relative to mean values should be studied for cross-parameters comparison. Third, the relationship between the magnitude of systematic error and PD patient characteristics has not been elucidated with regard also to unknown algorithm used for G-Walk system.

## Conclusions

This study documents the agreement between 8 gait parameters obtained from GAITRite and G-Walk in a group of healthy volunteers and PD patients. Stride duration and cadence exhibited the highest level of agreement of all the tested parameters. On the contrary, the duration of the gait cycle phases showed a lower level of agreement.

The final results of this study indicate that the G-Walk system can be used for evaluating the gait characteristics of the healthy subjects as well as the PD patients, with the exception of the phases of the gait cycle, which should be used with caution due to a systematic error.

##  Supplemental Information

10.7717/peerj.8835/supp-1 Supplemental Information 1 Gait parameters of healthy subjects and Parkinson’s disease patients

## Additional Information and Declarations

### Data Availability

The following information was supplied regarding data availability: The raw measurements are available as a Supplemental File.
